# Green Light Mitigates Cyclic Chronic Heat-Stress-Induced Liver Oxidative Stress and Inflammation via NF-κB Pathway Inhibition in Geese

**DOI:** 10.3390/antiox13070772

**Published:** 2024-06-27

**Authors:** Binbin Guo, Leyan Yan, Yi Tang, Jie Du, Zichun Dai, Jie Liu, Mingming Lei, Zhuocheng Hou, Huanxi Zhu

**Affiliations:** 1 Key Laboratory for Crop and Animal Integrated Farming, Ministry of Agriculture and Rural Affairs, Institute of Animal Science, Jiangsu Academy of Agricultural Sciences, Nanjing 210014, Chinayanleyan0710@jaas.ac.cn (L.Y.); 2212217039@stmail.ujs.edu.cn (Y.T.); 20210064@jaas.ac.cn (Z.D.); 20230104@jaas.ac.cn (J.L.); 20140036@jaas.ac.cn (M.L.); 2 Jiangsu Province Engineering Research Center of Precision Animal Breeding, Institute of Animal Science, Jiangsu Academy of Agricultural Sciences, Nanjing 210014, China; 3 School of Life Science, Jiangsu University, Zhenjiang 212000, China; 4 Animal Husbandry and Veterinary College, Jiangsu Polytechnic College of Agriculture and Forestry, Jurong 212400, China; dujiekycg@163.com; 5 College of Animal Science and Technology, China Agricultural University, Beijing 100193, China

**Keywords:** monochromatic light, geese, antioxidant capacity, inflammation, NF-κB signalling

## Abstract

Heat stress (HS) induces various physiological disorders in poultry, negatively impacting feed intake, feed efficiency, and growth performance. Considering the documented anti-stress and growth-promoting benefits of monochromatic green light in poultry, we aimed to investigate its effects on cyclic chronic HS-induced oxidative stress (OS) and inflammation in geese. We established three treatment groups—geese exposed to white light (W), white light with HS treatment (WH), and green light with HS treatment (GH)—treated over a six-week period with daily HS sessions. The results revealed that cyclic chronic HS induced liver OS and inflammation, leading to hepatocellular injury and reduced growth performance and feed intake. In comparison, the growth performance of geese under green light significantly improved. Additionally, liver index, serum, liver malondialdehyde (MDA), interleukin-6 (IL-6), interleukin-8 (IL-8), and tumour necrosis factor-α (TNF-α) levels were reduced. Serum total antioxidant capacity (T-AOC), liver catalase (CAT), and superoxide dismutase (SOD) activity were enhanced, reducing hepatic OS and inflammation. Liver transcriptomic analysis indicated that green light alleviates cyclic chronic HS-induced liver injury and promotes geese growth performance by suppressing NF-κB pathway activation.

## 1. Introduction

With the increasing severity of global warming, heat stress (HS) has become a prevalent environmental threat to animal husbandry [1,2]. HS occurs when the body is exposed to an ambient temperature that exceeds the upper limit of the isothermal zone [3]. Poultry is particularly vulnerable to HS due to their feather coverage, limited heat loss capacity, and lack of sweat glands [4,5,6]. HS can cause a series of physiological disorders in poultry, including systemic immune disorders, endocrine disorders, respiratory alkalosis, and electrolyte imbalance, which can adversely affect feed intake, feed efficiency, and growth performance [7,8].

Multiple studies have linked the decline in growth performance under HS to oxidative stress (OS) and inflammatory damage [9,10,11]. The liver, known as the centre of metabolism, is highly sensitive to abnormal temperature changes and is a major target for tissue damage during HS [12]. When HS occurs, the liver increases energy mobilisation and catabolism of proteins and carbohydrates to cope with the increased energy demand of the body, which seriously affects the accumulation of substances and energy required for growth [5,13]. Additionally, the accelerated metabolism in the liver leads to excessive generation of reactive oxygen species (ROS) in the mitochondria, causing OS and impairing normal liver function [14,15]. However, research on HS has mainly focused on pigs, chickens, cattle, and other animals [13], with limited studies on geese. Geese, with their fine, soft plumage and large fluff, are susceptible to HS [16]. In the hot summer of China, the economic loss due to cyclic chronic HS for each goose is more than CNY 10. Therefore, it is of great practical significance to alleviate the impact of cyclic chronic HS on meat geese.

Various methods have been employed to reduce the damage caused by HS, including improving the ventilation and cooling of breeding houses, adjusting feed formulation and feeding methods, and using feed additives [6,10,17]. Poultry are highly sensitive to light due to their unique visual structures and developed visual function [18,19], and they exhibit different stress susceptibilities under varying light spectra [20]. Therefore, ambient lighting management has attracted considerable attention in poultry farming, particularly the application of monochromatic light.

Studies have demonstrated that blue and green LED lights can reduce lipid peroxidation, improve antioxidant status, and enhance meat quality compared with red and white light [21,22]. Blue or green LED lights have shown potential in reducing the fear stress response in ducks and broilers [23,24]. Additionally, blue or green light improves immune performance by promoting T lymphocyte proliferation, antibody production, and humoral immunity [25,26]. Behavioural studies have also indicated that birds exhibit a distinct light colour preference under HS [27,28]. When exposed to different LED lights, broilers and ducks under green light show higher daily body weight gain and market body weight [29,30]. The probable mechanism is that light information acting directly on the retina affects the expression of arylalkylamine N-acetyltransferase (*AANAT*), a key regulator of melatonin biosynthesis [31,32], further regulating the growth performance and immune function of birds. Shorter wavelengths of light are more likely to cause retinal damage [33]. Given the multiple effects of green light on relieving stress, improving antioxidant capacities, and promoting growth performance, we hypothesised that green light has potential applications in alleviating HS in geese. However, the mechanisms of monochromatic green light in alleviating the harmful effects of HS and the involvement of the liver in this process remain unclear.

Therefore, we aimed to elucidate the effects of monochromatic green light on cyclic chronic HS in farmed geese and conducted a liver transcriptomic analysis to reveal the underlying molecular mechanisms.

## 2. Materials and Methods

### 2.1. Animal Ethics

All procedures used in this study were approved by the Research Committee of Jiangsu Academy of Agricultural Sciences (IACUC number: NKYVET 2014-63). All methods and management procedures complied with the *Regulations for the Administration of Affairs Concerning Experimental Animals*, approved by the State Council of the People’s Republic of China.

### 2.2. Animals and Treatment Design

A total of 144 Yangzhou ganders at 20 d of age (884.2 ± 23.0 g) were randomly divided into three environmental chambers in the Poultry Farm of the Luhe Animal Science Base of Jiangsu Academy of Agricultural Sciences (118°62′ E, 32°48′ N) in Jiangsu, China, with dimensions of 4 m × 2.5 m × 2.6 m (length × width × height). The initial body weights of the three groups were 879.8 ± 43.7 g, 886.5 ± 50.7 g, and 886.3 ± 51.6 g, respectively, without differences between groups. Each environmental chamber included three replicates, and each replicate housed 16 geese. From d 20 to d 45, the stocking density was 8 geese/m^2^, which was subsequently reduced to 5 geese/m^2^, and continued until the end of the experiment. All geese were raised on polyethylene prefabricated leakage dung plates. The floor was 60 cm above the ground, and about 20 cm thick rice husks were spread on the ground.

**Figure 1:**
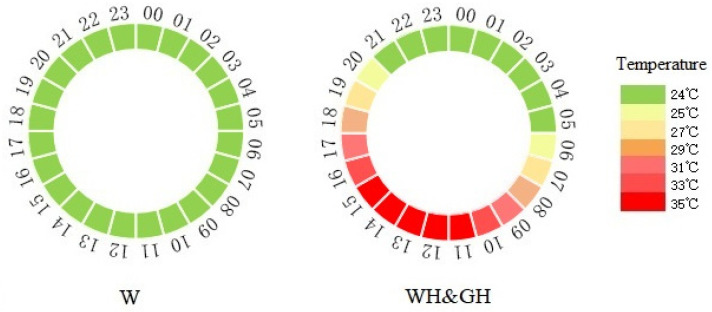
Light and temperature conditions for white light (W) group, white light with heat stress treatment (WH) group, and green light with heat stress treatment (GH) group. Numbers outside the circle represent time, and different colours of the circle indicate the temperature. In the W group, the daily ambient temperature was maintained at 24 °C, while in the WH and GH groups, the daily temperature was gradually increased to 35 °C from 06:00 to 11:00, maintained for 5 h, and then gradually decreased to 24 °C.

The relative humidity of each chamber was set to 70%. From d 21 to d 27, the temperature of the white light group (W), white light with high-temperature treatment group (WH), and green light with high-temperature treatment group (GH, 530–550 nm) was set to 24 °C. The W group was then maintained at 24 °C and the WH and GH groups were subjected to periodic high temperatures that mimicked an environmental heat wave (Figure 1) until the geese reached 70 days of age. The temperature that induces HS was determined by behavioural observations of geese. The temperature setting was feasible when panting was observed in most of the geese. Temperature in both houses as maintained by a computerized system controlling heaters and ventilation fans. All light sources were equalised at an illuminance of 100 lx at the geese’s head level using Testo 540 illuminometer (Testo SE & Co. KGaA, Lenzkirch, Germany) with a 16 h light/8 h dark cycle (dark from 22:00 to 06:00).

The geese had free access to feed and water. Drinking water was provided through an automatic drinking system, and the height of the drinking system was adjusted according to the geese’s height. Each replicate was equipped with a 10 kg feeding bucket. The nutritional composition of diets at different age stages was the same as that described by Guo et al., 2022 [34].

### 2.3. Growth Performance and Tissue Weight

The body weight (BW) of the geese was measured on d 28 and d 70. The average daily feed intake (ADFI) was recorded by subtracting the weight of the leftover feed from that of the feed supplied, and the spilled feed was ignored. The feed conversion ratio (FCR) was determined as the ratio of feed consumed to body weight gained. On d 70, eight geese from each group were randomly selected and slaughtered by decapitation after blood collection. The weight of abdominal fat was recorded, and the relative weight of abdominal fat (RWAF) was calculated by dividing the abdominal fat weight by the live body weight of the goose. Liver weight (LW) was recorded and expressed as absolute liver weight and liver index (relative to live body weight).

### 2.4. Blood Sampling and Analysis

Blood samples were collected and centrifuged for 15 min at 3000× *g*, 4 °C. Serum was separated for the measurement of glutamic pyruvic transaminase (GPT), glutamic oxaloacetic transaminase (GOT), corticosterone (CORT), antioxidant enzyme activity, and inflammatory factors.

The activity of GPT, GOT, superoxide dismutase (SOD), and catalase (CAT); concentrations of malondialdehyde (MDA); and the total antioxidant capacity (T-AOC) in serum were assessed using commercial kits (Nanjing Jiancheng Bioengineering Institute, Nanjing, China), according to the manufacturer’s instructions. Serum cytokines, including interleukin-1 beta (IL-1β), interleukin-6 (IL-6), interleukin-8 (IL-8), and tumour necrosis factor-α (TNF-α) levels, were assayed using ELISA kits supplied by MLBio (Shanghai, China), with intra- and inter-assay coefficients of variation less than 10% and 15%, respectively. Each sample was tested in triplicate.

### 2.5. Liver Sampling

Liver tissues (*n* = 8) were promptly sampled and cooled in liquid nitrogen to preserve their integrity for subsequent analyses, including assessments of antioxidant capacity, inflammatory factors, and transcriptomics. A subset of liver samples was fixed in 4% paraformaldehyde for histopathological analyses.

### 2.6. Histomorphology and Histopathology Analyses of Liver

Following a 24 h fixation period, liver tissues underwent dehydration, embedding, and sectioning into 5 µm thick paraffin sections. Subsequent dewaxing and dehydration were performed before staining with Hematoxylin and Eosin (H&E) (Wuhan Servicebio Technology Co., Ltd., Wuhan, China). Microscopic imaging was conducted using a light microscope (Olympus BX50, Tokyo, Japan) equipped with Olympus XC10 microdigital camera (Olympus Corporation, Tokyo, Japan) to observe morphological and histopathological changes at 400× magnification.

### 2.7. Liver Tissue Homogenate Preparation and Parameter Determination

Liver tissues (*n* = 8) were homogenized in ice-cold PBS (pH 7.4) and subjected to centrifugation (2000× *g* for 10 min at 4 °C) to extract supernatants. These supernatants were stored at −20 °C for subsequent assays of antioxidant enzyme activity (SOD and CAT), MDA and T-AOC concentrations, and cytokine levels (IL-1β, IL-6, IL-8, and TNF-α).

### 2.8. Transcriptomic Analysis

Total RNA was extracted from liver tissues (four biological replicates per group) using a TRIzol reagent kit (Invitrogen, Carlsbad, CA, USA). Following quality assessment and enrichment via oligo (dT) beads, mRNA was reverse-transcribed into cDNA using the NEBNext Ultra RNA Library Prep Kit for Illumina (NEB#7530, New England Biolabs, Ipswich, MA, USA). Purified double-stranded cDNA fragments underwent end-repair, the addition of a base, and ligation to Illumina sequencing adapters. This was followed by purification, size selection, and PCR amplification. Sequencing was performed using an Illumina Novaseq6000 (Gene Denovo Biotechnology Co., Guangzhou, China), and reads were mapped to the Anser cygnoides genome (GCF_000971095.1).

Gene expression was quantified as fragments per kilobase of transcript per million mapped reads. Principal component analysis (PCA) was performed using the R package g models (http://www.rproject.org/) between the W and WH groups and between the WH and GH groups. The differential expression analysis was conducted using DESeq2 (v1.30.1). Genes with *p* value below 0.05 and fold change ≥1.5 or ≤0.5 were considered differentially expressed genes (DEGs). Functional annotation enrichment analyses of Gene Ontology (GO) and *Kyoto Encyclopedia of Genes and Genomes* (KEGG) terms were conducted using the online platforms http://www.Geneontology.org/ and http://www.genome.jp/, respectively. GO terms and KEGG pathways with FDR ≤ 0.05 were identified through functional annotation enrichment analyses.

### 2.9. qRT-PCR Analysis

Total RNA extracted for RNA sequencing was used for cDNA synthesis using the Prime Script RT reagent kit with the gDNA Eraser (Perfect Real Time) (TaKaRa Biotechnology Dalian Co., Ltd., Dalian, China). qRT-PCR was performed using TB Green^®^ Premix Ex Taq™ II (TliRNaseH Plus) (TaKaRa Biotechnology Dalian Co., Ltd., Dalian, China), and relative expression levels were calculated via the 2^−ΔΔCt^ method. Expression values were compared with RPKM values of RNA-seq, with GAPDH serving as an internal control. Primers used for qRT-PCR are listed in Table 1.

### 2.10. Statistical Analysis

Data are shown as means ± SEM and were analysed using Prism v8.0 with one-way ANOVA followed by Tukey’s multiple comparison test. Differences were considered statistically significant at *p* < 0.05.

## 3. Results

### 3.1. Behaviour, Growth Performance, and Tissue Weight

When the ambient temperature was maintained at 35 °C, panting and reduced activities were observed in the majority of geese in heat-treated groups.

Table 2 illustrates the growth performance and tissue weight outcomes following 42 days of cyclic chronic temperature exposure. Geese in the W and GH groups exhibited similar final body weights, which were significantly (*p* < 0.05) higher than those in the WH group. Likewise, the RWAF mirrored the trend in goose body weight. The ADFI was significantly (*p* < 0.05) lower in the WH group compared to the W group, and the FCR was significantly (*p* < 0.05) higher. Compared with the WH group, ADFI in the GH group had a tendency to increase and showed no differences with the W group. However, FCR in the GH group decreased significantly (*p* < 0.05) compared to the W group. LW did not differ significantly among the three groups, although the liver index of geese in the WH group was significantly higher than those in the W and GH groups (*p* < 0.01). No substantial differences were observed in the ADFI, FCR, and liver index between the W and GH groups.

### 3.2. Liver Morphology and Histopathology

**Figure 2:**
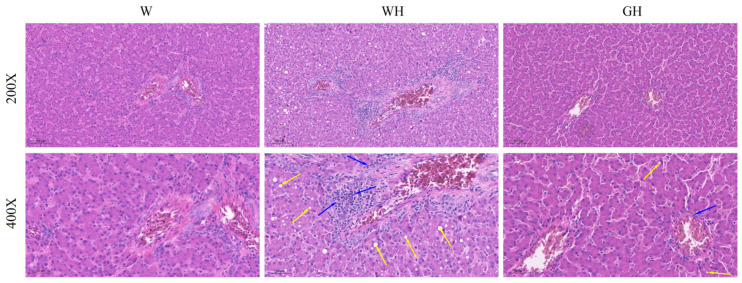
Effect of different light and temperature treatment on liver histopathological changes in geese. Inflammatory infiltration and aggregation are indicated by blue arrows and steatosis by yellow arrows.

Upon morphological analysis post-H&E staining (Figure 2), hepatocytes in the W and GH groups exhibited a neatly arranged structure with clear boundaries, uniform cytoplasm, and a pink colouration. The hepatic cords were distinctly delineated and arranged in a scattered pattern. Conversely, in the WH group, hepatic cord arrangement was disrupted, and the cytoplasm appeared light pink and uneven. Further histopathological examination revealed inflammatory cell infiltration and aggregation (marked with blue arrows) around blood vessels in the livers of geese in the WH group. In contrast, the W and GH groups showed minimal inflammatory cell infiltration. Additionally, numerous round vacuoles of varying sizes were observed in the WH group (marked with yellow arrows).

### 3.3. Serum Corticosterone and Liver Tests

As indicated in Table 3, cyclic chronic HS significantly increased CORT levels in the serum of the WH (*p* < 0.01) and GH (*p* < 0.05) groups. However, green light exposure significantly (*p* < 0.05) reduced CORT levels compared to the WH group. Furthermore, cyclic chronic HS induced higher (*p* < 0.05) serum GPT and GOT levels in comparison to the W and WH group. Compared to WH, green light exposure mitigated the increases in serum GPT (*p* < 0.05) and GOT levels induced by cyclic chronic HS. No significant differences were observed in serum GPT and GOT levels between the W and GH groups.

### 3.4. Antioxidant Parameters in Serum and Liver

**Figure 3:**
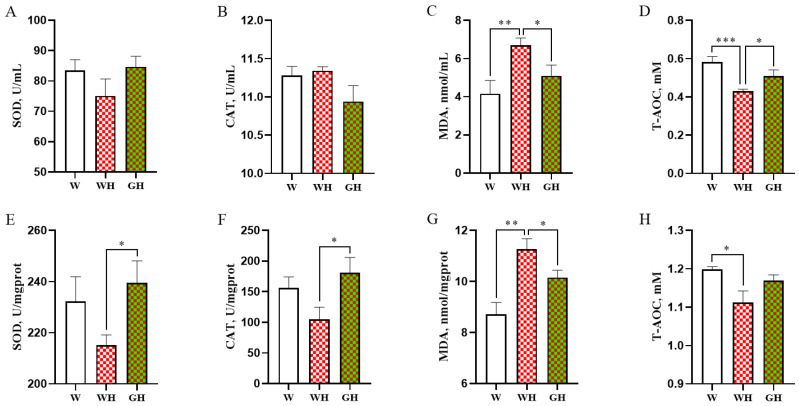
Effects of different light and heat exposures on the antioxidant enzyme activity of superoxide dismutase (SOD), glutathione peroxidase, and catalase (CAT), as well as an increase in the production of malondialdehyde (MDA) levels and total antioxidant capacity (T-AOC). (**A**–**D**) In serum. (**E**–**H**) In liver. Data are expressed as means ± SEM, *n* = 8. * represents *p* < 0.05, ** represents *p* < 0.01, and *** represents *p* < 0.001.

The activities of antioxidant enzymes (SOD and CAT), levels of MDA, and the T-AOC in both serum and liver are depicted in Figure 3. Comparison between the W and WH groups revealed that cyclic chronic HS significantly (*p* < 0.01) increased MDA levels in both serum and liver, while T-AOC significantly (*p* < 0.05) decreased in the WH group. However, exposure to green light significantly (*p* < 0.05) reduced serum and liver MDA levels compared to the WH group, accompanied by enhanced serum T-AOC, liver SOD, and CAT activity (*p* < 0.05). No significant differences were observed in SOD and CAT activity, MDA levels, and T-AOC in both serum and liver between the W and GH groups.

### 3.5. Inflammatory Parameters in Serum and Liver

**Figure 4:**
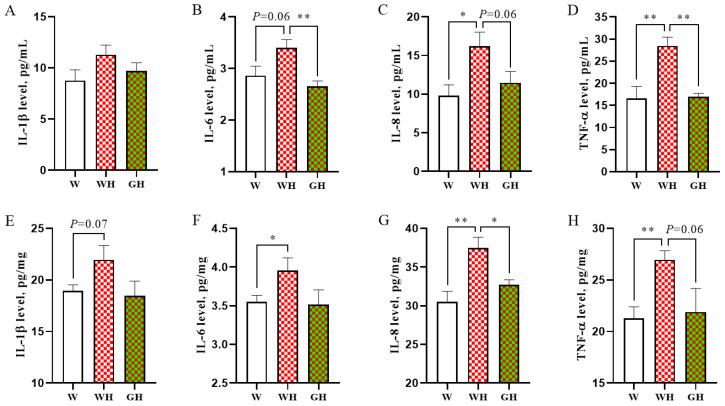
Effects of different light and heat exposures on inflammatory parameters in serum and liver. (**A**–**D**) Serum interleukin-1 beta (IL-1β), interleukin-6 (IL-6), interleukin-8 (IL-8), and tumour necrosis factor-α (TNF-α) levels, respectively. (**E**–**H**) Liver IL-1β, IL-6, IL-8, and TNF-α levels, respectively. Data are expressed as means ± SEM, *n* = 8. * represents *p* < 0.05, and ** represents *p* < 0.01.

In Figure 4, the inflammatory parameters in both serum and liver are delineated. Comparison between the W and WH groups revealed a significant increase in serum IL-8 (*p* < 0.05) and TNF-α levels (*p* < 0.01) due to cyclic chronic HS, with IL-6 levels showing a tendency to increase. Notably, in the liver, IL-8 and TNF levels exhibited the most significant increases (*p* < 0.01), followed by IL-6 levels (*p* < 0.05). Transitioning from white light under HS to green light resulted in varied degrees of decrease in IL-6, IL-8, and TNF-α levels in both serum and liver, with the reductions in serum IL-6 and TNF-α being the most prominent (*p* < 0.01). No significant differences were observed between the W and GH groups for these parameters.

### 3.6. Liver Transcriptome Reveals the Potential Molecular Regulatory Mechanism of Monochromatic Green Light in Alleviating Heat Stress

#### 3.6.1. mRNA Transcriptomes of Liver in W, WH, and GH Group

**Figure 5:**
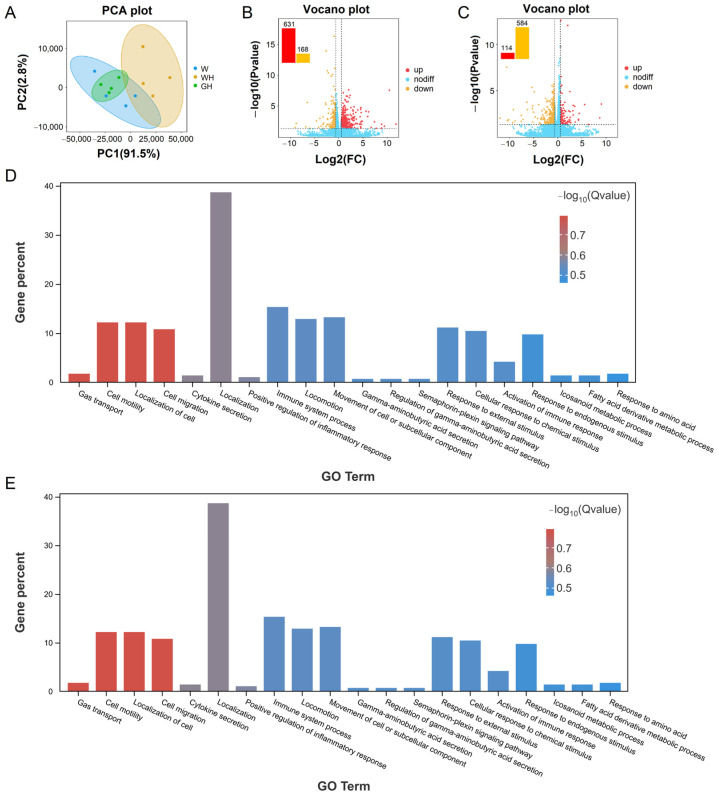
Effect of different light and heat exposures on transcriptomics profiles in goose liver. (**A**) Principal Component Analysis (PCA) indicated that, while the W and GH groups are similar, the WH group is distinctly different from the other two groups. Volcano plots (**B**,**C**) indicate significant DEGs in the livers of the W, WH, and GH groups. Red dots (up) represent significantly up-regulated genes (log2FC > 0.585, *p* value < 0.05); orange dots (down) represent significantly down-regulated genes (log2FC < 20.585, *p* value < 0.05); blue dots (no diff.) represent no significant differences. (**D**,**E**) Top 20 enriched categories of ontology (GO) terms of the DEGs. The x axis shows the GO category (biological process), and the y axis represents the percent of genes.

PCA results demonstrated a significant separation between the W and WH groups, as well as between the WH and GH groups (Figure 5A). Further analysis revealed 799 DEGs between the W and WH groups, comprising 631 up-regulated and 168 down-regulated genes (Figure 5B). Similarly, 698 DEGs (114 up-regulated and 584 down-regulated) were identified in the WH and GH groups (Figure 5C).

#### 3.6.2. Enrichment Analysis of DEGs

The GO annotations of these DEGs showed enrichment in 52 GO terms for both the comparison of W versus WH and WH versus GH. The top 20 significantly enriched GO terms were predominantly related to the BP. Compared to the W and WH groups, significantly enriched GO terms included amino acid metabolic processes, organic acid metabolic processes, immune system processes, and long-chain fatty acid metabolic processes (Figure 5D). DEGs involved in these processes included *GPT2*, *TLR2*, and *CCL2*, among others (Table S1). Conversely, compared to the WH and GH groups, significantly enriched GO terms encompassed gas transport, cell motility and localisation, cytokine secretion, regulation of inflammatory response, and immune system processes. DEGs involved in these processes included *CCR7*, *NFKBIA*, and *HSP90AA1*, among others (Table S2). These findings suggest that chronic cyclic HS predominantly affects liver immune and metabolic functions, and compared to the WH group, monochromatic green light may influence biological changes such as inflammation, immunity, and cell localisation.

**Figure 6:**
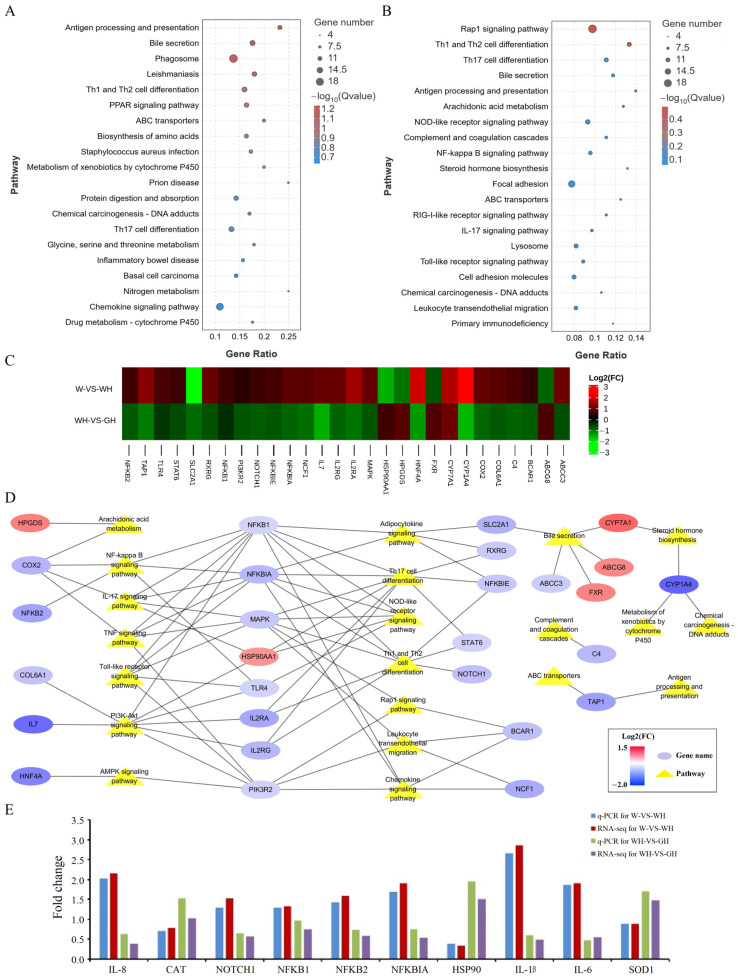
Potential pathway analysis of green light in regulating liver heat stress responses. (**A**) Top 20 significantly enriched KEGG pathways of DEGs in comparison between W and WH groups. (**B**) Top 20 significantly enriched KEGG pathways of the DEGs in comparison between WH and GH groups. (**C**) Heatmap of Log2(FC) of 28 DEGs related to immunity and inflammation presented in top 20 pathways in both comparisons. (**D**) Gene–pathway network diagram in the comparison of WH and GH groups. Blue and red ellipses represent significantly up-regulated genes (log2FC > 0.585, *p* < 0.05) and down-regulated genes (log2FC < 20.585, *p* < 0.05), respectively; yellow triangles represent pathways. (**E**) Validation of the gene expression profile by qRT PCR (*n* = 8).

To further elucidate crucial biochemical metabolic pathways and signal transduction pathways related to DEGs, KEGG enrichment analysis was performed for the comparisons of W versus WH and WH versus GH. The top 20 KEGG pathways and the ratios of genes involved in these pathways are illustrated in Figure 6A and Figure 6B, respectively. Pathways affected by chronic cyclic HS included antigen processing and presentation, th1 and th2 cell differentiation, th17 cell differentiation, inflammatory bowel disease, chemokine signalling, bile secretion, protein digestion and absorption, PPAR signalling, and nitrogen metabolism, indicating a predominant impact on liver immune function, inflammatory response, and metabolism (Figure 6A). Transitioning from white light to green resulted in DEGs predominantly enriched in signal transduction, immunity and inflammation, the digestive system, and metabolism (Figure 6B).

To unravel the potential molecular regulatory mechanism of green light in alleviating cyclic chronic HS-induced liver OS and inflammatory response, 28 DEGs related to immunity and inflammation present in the top 20 pathways in both comparisons were identified. A gene–pathway network was mapped in the comparison of WH and GH. The heatmap of Log2(FC) (Figure 6C) depicted opposite expression trends of these DEGs in both comparisons. The gene–pathway network diagram (Figure 6D) highlights the critical role of the NF-κB signalling pathway in the inflammatory response during green light and cyclic chronic heat exposure. Transcription factors *NFKB1*, *NFKB2*, and *NFKBIA* were involved in regulating many other immunity and inflammation pathways, including the IL-17 signalling pathway, TNF signalling pathway, adipocytokine signalling pathway, Toll-like receptor signalling pathway, th17 cell differentiation, th1 and th2 cell differentiation, NOD-like receptor signalling pathway, and chemokine signalling pathway. Expression levels of these three genes were significantly down-regulated in the GH group compared to the WH group, indicating negative regulation of inflammatory responses.

#### 3.6.3. Quantitative Real-Time PCR Validation of mRNAs

To validate the sequencing data, ten genes selected from the RNA-seq expression profiles underwent verification by qRT-PCR. The results demonstrated excellent consistency between the RNA-seq and qRT-PCR verifications, affirming the reliability of our data and the transcriptome sequencing platform (Figure 6E).

## 4. Discussion

This study evaluated the protective effect of green light on geese under cyclic chronic HS and explored the potential molecular regulatory mechanisms. Cyclic chronic HS induced OS and inflammation in goose liver, leading to hepatocellular injury and reduced growth performance. Monochromatic green light had a “protective effect” under HS compared to white light. Further transcriptome analysis revealed that green light inhibited NF-κB signalling, resulting in reduced hepatic OS and inflammation.

Poultry under HS conditions spend more time resting and panting and less time feeding and walking [35]. In our study, upon increasing the ambient temperature to 35 °C, panting and reduced activities were observed in the majority of geese in the heat-treated groups, confirming successful induction of HS. Additionally, circulating CORT is commonly regarded as a reliable indicator of heat stress in poultry [14,36]. Consistently, our study demonstrated a significant increase in serum CORT levels following cyclic chronic high-temperature treatment for 6 weeks. Moreover, our findings indicated that cyclic chronic HS significantly reduced geese market BW and ADFI, resulting in a higher FCR. Conversely, in comparison to the W group, these parameters were not significantly affected by HS in the green light illumination group, suggesting the potential of green light to mitigate HS effects.

Researchers have long focused on investigating the effects of monochromatic light on the growth performance of poultry. Short-wavelength light can influence poultry behaviour and promote growth by stimulating the somatotropic axis [37]. Broilers raised under green light exhibited increased feeding and drinking time, leading to enhanced feed intake [38,39]. Additionally, the height of intestinal villi and the number of goblet cells in the small intestine of broilers under green light illumination were higher than those under white light illumination [40]. Longer villi and shallower crypt depths facilitate the digestion and absorption of nutrients [41], while goblet cells contribute to mucosal surface protection and local intestinal immune regulation, promoting intestinal health and animal growth [42]. Studies on broilers have demonstrated that chronic heat exposure impairs intestinal morphology and appetite, consequently reducing growth performance [43,44]. The comparison between WH and GH groups suggests that the increased ADFI and improved feed utilization in the green light illumination group align with the observed increase in market body weight of meat geese. Although the ADFI of meat geese in the GH group was only about 2% higher than that in the WH group, the final body weight of geese in the GH group was significantly higher, indicating that the increased BW and decreased FCR may, at least partially, result from improved intestinal development.

Previous research indicates that chronic HS leads to a reduction in fat content in birds under free-feeding conditions. For instance, Arbor Acres broilers exposed to HS exhibited significant decreases in subcutaneous and intermuscular fat deposits, along with slight reductions in abdominal fat [45]. Similarly, Smith [46] also noted a significant decrease in abdominal fat deposition in broilers subjected to cycling high temperatures. Consistent with these findings, our study observed a significant reduction in RWAF in geese experiencing cyclic chronic HS. On the other hand, an enhanced fat deposition was observed in birds under chronic heat exposure conditions [42]. The differences in the results above may be due to differences in the duration of HS. According to the results of liver morphology and histopathology, numerous round vacuoles of varying sizes were observed in the WH group, suggestive of potential fatty vacuole degeneration in the liver. Previous studies have shown that, in addition to impaired gut function [43,44], sustained HS might overwhelm the ability of the liver to export lipids, lead to liver lipid accumulation, and increased liver weight [47,48], which, in turn, reduced the liver-derived fatty acids needed for fat synthesis by adipose tissue. However, compared to the WH group, the liver index and liver testes exhibited a tendency to recover in the GH group, indicating the beneficial effect of green light on liver health.

After digestion and absorption in the intestine, the liver plays a crucial role in metabolising most of the fat, protein, and sugar from the diet. Insufficient antioxidant capacity in the liver can result in metabolic disorders that impact animal growth [49]. To further investigate the physiological mechanisms by which green light alleviates HS-induced growth retardation in meat geese, we focused on the liver, which is highly sensitive to HS [12] and a key metabolic site in the body [50]. Our results demonstrate that cyclic chronic HS reduces antioxidant capacity and damaged liver cells. When compared with WH, exposure to green light increased serum and liver SOD levels and reduced MDA concentrations. Previous research in chick embryos has shown that green light enhances liver antioxidant and anti-inflammatory capacity and promotes embryo growth via melatonin and its receptors [51]. Similarly, green-light irradiation in obese mice induced by a high-fat diet has been found to enhance liver antioxidant and anti-inflammatory capacity, thus restoring liver lipid metabolism function and maintaining normal body weight [52]. Additionally, dietary supplementation with phytochemicals possessing antioxidant properties has been shown to alleviate liver oxidative damage by enhancing liver antioxidant capacity [53,54]. Collectively, these findings suggest that, like other antioxidants, green light may mitigate the adverse effects of HS on goose growth by reducing oxidative stress in the liver.

Numerous studies have indicated that liver injury results from an inflammatory response mediated by oxidative damage [55,56,57]. In addition to enhancing liver antioxidant capacity, green light also alleviated liver inflammation, as evidenced by reductions in pro-inflammatory cytokines and attenuated inflammatory infiltration in the green light group. This effect has also been demonstrated in an obese murine model induced by a high-fat diet [52]. However, it is important to note that while some studies support the positive effects of green light on anti-inflammation and antioxidation, others suggest that blue light may alleviate HS in chicken embryos or broilers by enhancing liver antioxidant and anti-inflammatory capacity [21,58]. These mixed results may be attributed to differences in light treatment, such as colour, intensity, and photoperiod, as well as variations in animal species. Overall, our results indicate that green light has the potential to enhance the antioxidant and anti-inflammatory capacity of animals, thereby alleviating the pathological damage to the liver caused by HS.

GO enrichment analysis indicated significant alterations in amino acid metabolism, organic acid metabolism, and immune system processes due to HS. For instance, the glutamate pyruvate transaminase 2 (*GPT2*) gene, found in the top 5 enriched GO terms, was up-regulated by HS. *GPT2* encodes alanine transaminase, facilitating the reversible transamination between alanine and 2-oxoglutarate to produce pyruvate and glutamate. Under prolonged high temperatures, amino acids are diverted from tissue growth to metabolic energy production, contributing to liver gluconeogenesis [59,60]. This intensified liver metabolism leads to excessive ROS generation, resulting in liver cell damage and elevated aminotransferase levels in the blood [14,15]. Correspondingly, our study observed increased serum GPT and GOT activities in the HS-exposed group. Moreover, up-regulated genes under HS, such as *TLR2* and *CCL2*, can mediate immune responses and trigger inflammation [61], corroborating our findings. In the study of humans, it has been found that the levels of pro-inflammatory cytokines in blood were significantly up-regulated after heat stroke [62], and excessive inflammatory response is considered to be an important pathological mechanism of liver injury caused by HS [63]. In our study, the levels of IL-8 and TNF-α secreted by Th1 cells was significantly up-regulated by HS both in serum and liver, suggesting that Th1 cells were over-activated. Clinically, excessive activation of Th1 cells can lead to immune diseases, such as autoimmune hepatitis [64], suggesting that HS may cause immune diseases. In the comparison between HS and GH groups, enriched GO terms included cytokine secretion, immune system processes, and positive regulation of inflammatory responses, highlighting the role of green light in modulating liver immune and inflammatory responses under HS conditions.

Further KEGG enrichment analysis between WH and GH groups revealed that the NF-κB signalling pathway may be pivotal in green light’s mitigation of HS-induced liver oxidative stress and inflammatory responses. NF-κB is a redox-sensitive transcription factor implicated in DNA transcription regulation [49,53], cellular physiological changes, and responses to stress and free radicals [65]. Normally residing in the cytoplasm, the NF-κB complex translocate to the nucleus upon activation, triggering the transcription of target genes and amplifying inflammation [54]. Our results demonstrated that green light significantly inhibited the mRNA expression of *NFKB1*, *NFKB2*, and *NFKBIA* compared to the HS-exposed group, reducing the release of inflammatory cytokines. Excessive secretion of TNF-α and IL-6, activated by the NF-κB signalling pathway, is central to the pathophysiology of the inflammatory response [49,66], aligning with our inflammatory parameters and liver histopathology results.

Prior research has indicated that green light can stimulate the transcription of *AANAT*, a key enzyme regulating melatonin synthesis, consequently increasing melatonin secretion [31,67]. Melatonin, an endogenous indoleamine, and its metabolites exhibit potent antioxidant properties, directly scavenging ROS or activating antioxidant defence systems [68,69]. Studies on HS have demonstrated melatonin’s ability to alleviate HS-induced oxidative stress [70]. Melatonin enhances the expression of heat shock protein 90 (HSP90) via membrane receptors, counteracting HS’s inhibitory effect [58,71]. Moreover, melatonin inhibits NF-κB expression in liver tissue, reducing pro-inflammatory cytokine production [72]. HSP90, in turn, mitigates tissue inflammation via TLR4/NF-κB signalling pathway inhibition [73]. Notably, the liver accumulates high melatonin concentrations and serves as the primary site for melatonin metabolism [74]. In our study, chronic HS down-regulated the liver *HSP90* expression level, as observed by Safaa E. Abdo et al. [58], while green light significantly up-regulated it. Serum melatonin concentration (Figure S1) displayed a similar trend to that of *HSP90*. This evidence suggests that monochromatic green light may inhibit the NF-κB signalling pathway by boosting melatonin secretion and activating HSP90 transcription, thereby alleviating HS-induced liver inflammation and oxidative stress. However, further in vivo and in vitro tests are needed to demonstrate the inhibitory effect of melatonin and HSP90 on the NF-κB signalling pathway.

## 5. Conclusions

In summary, chronic cyclic HS induces OS and inflammatory damage in the livers of geese, impacting growth performance. According to our results, monochromatic green light may mitigate HS-induced liver injury and enhance goose growth performance by inhibiting the NF-κB signalling pathway through increased melatonin secretion and *HSP90* transcriptional activation. This mechanistic insight sheds light on the potential therapeutic role of green light in mitigating HS-induced liver injury and improving growth performance in geese.

## Figures and Tables

**Table 1:** Primers used for qRT-PCR.

Genes	Accession Number	Primer Sequence (5–3)	Fragment Size (bp)
*β* *-Actin*	XM_048058703.1	TGACGCAGATCATGTTTGAGA	159
		GCAGAGCGTAGCCCTCATAG	
*NFKB1*	XM_048066390.1	TGATTGCTGCTGGAGTTAATGT	172
		GCTGCTATGTGAAGAGGTGTT	
*NFKB2*	XM_048069036.1	CTTCACCGCCTACCTTCGT	172
		GCACAGCAAGTAGACCTCATC	
*NFKBIA*	XM_048059840.1	TCAGAAGCGTCAGCGTCCTCA	139
		AGCAGGTACTCGACAACAGCCA	
*IL-6*	XM_048070285.1	ATGTCGTCCGTCACTGTAGC	170
		GCGTGGAAGTAGCCTGAGAA	
*IL-8*	XM_013190618.2	AGGAAACTGGACTGCAGGGA	188
		TTGTGCCTGACTTGTGTGCT	
*NOTCH1*	XM_048053605.1	GCCTGTCCGAAGTGAACGAGTG	189
		TGTAGCCGCTGGTCATGTCCTT	
*IL-1* *β*	XM_048054159.1	CCCACAAAAGAAGCTTCGCC	172
		GAAGTCCTTGTGCGACGGC	
*HSP90α*	XM_048069885.1	GAGCGTCTTCGCAAACATGG	106
		CAGAAACCAGGGTCTTGCCT	
*CAT*	XM_048058871	GCGTATGCTGATACACATAGACATCGT	194
		CTCTCCTTCACAACAGGTTGATCTTCT	
*SOD1*	XM_048071453.1	TTACTGGAAGAATCAGCGGCTTGTC	136
		TCAGTTGGTCCACCGTGCTTCT	

**Table 2:** Growth performance and tissue weight of geese under different light and temperature treatments.

Items ^1^	Group			*p* Value
	W	WH	GH	
BW (d28), g	1775.1 ± 58.5	1790.8 ± 63.0	1803.4 ± 29.6	0.931
BW (d70), g	4038.3 ± 118.1 ^b^	3614.1 ± 132.1 ^a^	4013.3 ± 80.7 ^b^	0.031
ADFI, g	265.3 ± 5.0 ^b^	250.9 ± 3.1 ^a^	256.0 ± 1.3 ^ab^	0.049
FCR	4.90 ± 0.16 ^a^	5.76 ± 0.20 ^b^	4.84 ± 0.07 ^a^	0.029
RWAF, %	3.83 ± 0.23 ^b^	2.59 ± 0.30 ^a^	3.45 ± 0.17 ^b^	0.004
LW, g	71.57 ± 2.46	71.66 ± 3.19	67.64 ± 1.99	0.422
Liver index ^2^, %	1.70 ± 0.02 ^a^	1.94 ± 0.06 ^c^	1.69 ± 0.05 ^a^	0.006

^1^ BW, body weight; ADFI, average daily feed intake, FCR, feed conversion ratio; RWAF: relative weight of abdominal fat; LW, liver weight. ^2^ Calculated as the percentage of live body weight. Superscript letters in the same row denote significant (^a and b^: *p* < 0.05, ^b and c^: *p* < 0.05, and ^a and c^: *p* < 0.01) differences among groups.

**Table 3:** Effect of different light and heat exposure on geese serum corticosterone and liver tests.

Items ^1^	Group			*p* Value
	W	WH	GH	
CORT, ng/mL	117.55 ± 9.18 ^a^	175.96 ± 4.82 ^c^	148.12 ± 7.45 ^b^	<0.0001
GPT, U/L	68.32 ± 9.32 ^a^	103.8 ± 12.05 ^b^	72.88 ± 8.459 ^a^	0.049
GOT, U/L	80.55 ± 10.58 ^a^	133.70 ± 13.88 ^b^	112.10 ± 12.27 ^ab^	0.026

^1^ CORT, corticosterone; GPT, glutamic pyruvic transaminase; GOT, glutamic oxaloacetic transaminase. Superscript letters in the same row denote significant (^a and b^: *p* < 0.05, ^b and c^: *p* < 0.05, and ^a and c^: *p* < 0.01) differences among groups.

## Data Availability

Data is contained within the article and Supplementary Materials.

## Supplementary Materials

The following supporting information can be downloaded at: https://www.mdpi.com/article/10.3390/antiox13070772/s1, Table S1: Top 20 enriched GO terms and involved DEGs in comparison to W and WH group; Table S2: Top 20 enriched GO terms and involved DEGs in comparison to WH and GH group; Figure S1: Serum melatonin concentration of W, WH, and GH group, * represents *p* < 0.05.
