# Supplementary figures and images for: Green Light Mitigates Cyclic Chronic Heat-Stress-Induced Liver Oxidative Stress and Inflammation via NF-κB Pathway Inhibition in Geese
Source: Antioxidants (Basel). 2024 Jun 27;13(7):772. doi: 10.3390/antiox13070772 (PMC11274274; doi:10.3390/antiox13070772)

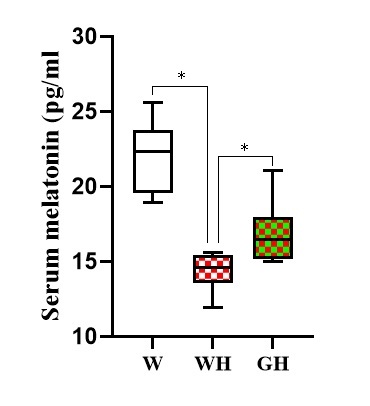

Supplement: Supplementary file 1 [file antioxidants-13-00772-s001.zip › Figure S1.jpg]
